# Supplementary material for: Are carbon emissions trading and green financial instruments synergistic? -Comprehensive quantitative research based on content analysis
Source: PLoS One. 2024 Mar 7;19(3):e0298601. doi: 10.1371/journal.pone.0298601 (PMC10919601; doi:10.1371/journal.pone.0298601)
Supplement: S1 Data — (ZIP) [file pone.0298601.s001.zip › Data/地级市Z_F工作报告.zip!/╝¬┴╓/░╫│╟╩╨/└┤╫╘╣1⁄2╓┌║┼í╛┬φ┐╦╩2╛▌═°í┐_2009.docx]

各位代表：

　　现在，我代表白城市人民政府，向市第四届人民代表大会第二次会议报告政府工作，请予审议，并请市政协各位委员提出意见。

　　一年来，在国家大事要事多、困难挑战大的环境下，我们按照省委、省政府的部署要求，在市委的正确领导下，深入贯彻落实科学发展观，带领全市广大干部群众奋力拼搏，推进白城崛起迈出重大步伐，全市经济社会发展呈现强劲势头。市四届人大一次会议审议批准的发展目标全面实现，重点目标实现了较大突破。一是地区生产总值增速加快。预计全市地区生产总值完成290亿元，同比增长20.1%，增速处于全省9个市(州)前3位。二是固定资产投资较快增长。预计全市固定资产投资完成262亿元，同比增长42.5%，超过省下达计划任务5.5个百分点。三是工业经济效益显著提高。在国际金融危机冲击、经济增速下滑的形势下，全市规模以上工业预计实现增加值37亿元，同比增长21%；实现利润6.2亿元，同比增长17%。四是财政收入增量和增幅均创历史新高。预计全市一般预算全口径财政收入完成22.3亿元，同比增长24%。市本级一般预算全口径财政收入预计完成7亿元，同比增长16.4%。其中地方级财政收入保持了20%以上增长。五是粮食产量达到历史最高水平。在人努力、天帮忙的情况下，预计全市粮食总产量达到62亿斤，比去年增产25.3亿斤。六是牧业经济发展突破历史最高点。预计全市牧业产值完成59亿元，同比增长43.9%；牧业人均收入达到1520元。特别是在“三鹿奶粉”事件影响下，我市奶业仍然保持较高发展势头，奶牛存栏达11万头，已占我省奶牛存栏的“半壁江山”。七是引进战略投资者实现重大突破。预计全市引进域外资金200亿元，同比增长25%，是增幅最高的一年。其中实际利用外资3740万美元，同比增长97.4%，增幅在全省处于第3位。外贸进出口完成10100万美元，在历史上首次突破1亿美元大关。目前，全市已有8户央企、3户世界500强企业、12户国内500强企业、3户跨国公司、14户上市公司入驻白城开发建设。八是城乡居民收入有较大增加。预计全市城镇居民人均可支配收入达到13450元，同比增长16.6%；农民人均纯收入达到3551元，同比增长24.6%。

　　今年以来，我们重点抓了以下八个方面工作：

　　（一）强化固定资产投资，事关白城长远发展的重大项目建设实现较大突破。今年，是我市引进千万元以上项目最多、重大项目建设进展最快的一年。全市启动建设千万元重点项目206个，总投资1248亿元。目前，已滚动发展250个项目，其中亿元以上建设项目77个，10亿元以上建设项目14个，共完成投资206亿元。特别是引进了投资20亿元的香港吉祥集团玉米深加工、投资3.5亿元的新加坡益海嘉里粮油综合加工等一批具有较强拉动作用的大项目；启动了投资19亿元的白城至石头井子高速公路等重大项目建设；加快了投资3亿元的盛吉蓖麻深加工、投资5亿元的天福公司20万头牛养殖屠宰加工、投资2.2亿元的雨润集团(镇赉)千万只鸭鹅养殖加工等项目建设进度；推进了洮北宏帝达汽车齿轮加工、洮南恒盛毛纺技术改造、大安石油机械生产等项目建设速度；促进了金升镍业、公益源黑大豆加工等重点项目建设竣工投产，为实现白城崛起奠定了坚实基础。

　　（二）加强能源基地建设，电力产业发展迅猛、位居全省前列。开工建设了谋划运作多年、投资50亿元的中电投白城发电公司一期工程2×66万千瓦火电项目，已累计完成投资12.6亿元；投资80亿元的二期工程2×100万千瓦项目预可研已通过专家评审。加快了投资10.6亿元的白城500千伏输变电工程项目建设进度；投资14亿元的白城热电厂扩建工程项目，审批手续基本完成，已经开始征地工作。相继开工建设了洮北宝山和华能二期、洮南大唐二期、镇赉德恒、大安中广核二期、通榆同发扩建和40万千瓦风电特许经营权等风电项目。目前，全市已建立风力发电场18个，立项装机达到128万千瓦，实现并网发电105万千瓦，占全国风力发电总和的10%。风电设备制造企业已发展到9个，初步形成了以风机、塔筒等部件为主导产品的风电设备制造产业。同时，启动建设镇赉石油增产、大安石油开发、洮南铜矿开采等项目，我市已成为吉林西部重要的能源城市。

　　（三）全力实施粮食增产工程，水利设施建设、土地开发整理实现历史性突破。紧紧抓住我省增产百亿斤商品粮机遇，启动实施了粮食增产“223”工程，就是力争用三年时间，新增水田面积200万亩，新增旱田全程灌溉面积200万亩，新增粮食产量30亿斤以上，使全市粮食生产能力提高到75亿斤阶段性水平。全市规划建设11个工程、22个项目，目前已相继开工建设。强化水利工程建设，较好完成了引嫩入白、大安灌区、洮儿河灌区、月亮泡蓄滞洪区、分洪入向“五大水利工程”年度建设计划。加快推进西部土地开发整理镇赉和大安“两大项目区”建设，启动建设镇赉、大安国家级生态农业示范区项目。加强标准粮田建设，实施“百万亩玉米保护性耕作示范推广工程”。发展循环经济，利用电厂脱硫废渣治理碱地试验取得了成功。加强“三大生态产业工程”建设，植桑面积达到55万亩，山杏面积达到104万亩，白刺面积达到50万亩。推进农业产业化，加快农业科技园区建设和庭院经济发展步伐。发展特色农业，绿豆、油葵、辣椒、燕麦、蓖麻、烤烟等形成了基地化种植、产业化发展的格局，现代烟草农业发展扎实推进。农畜产品加工业发展势头强劲，形成了水稻、玉米、葵花、蓖麻、辣椒、绿豆、燕麦、鲜奶、白鹅、狐貉、肉鸡等加工支柱产业发展框架。稳步推进新农村建设，启动省、市、县推进村119个，开工建设项目432个，完成投资19.3亿元。第二次土地调查工作深入推进。今年，不论是建设规模，还是资金投入，都是我市农田水利基本建设史上没有过的，全市农业发展迈上了一个新台阶。

　　（四）坚定不移地走工业园区化发展道路，工业经济发展能量进一步增强。白城工业园区实现了跨越发展，入园项目达到88个，已有39个项目开工建设，36个项目投产运营。今年，预计园区地区生产总值实现4.2亿元，同比增长334%；工业总产值实现15.2亿元，同比增长368%；工业增加值实现3.7亿元，同比增长382%；全口径财政收入实现2650万元，同比增长135%。各县(市)工业集中区建设加快推进，洮北龙丹乳业、镇赉银诺克药业、洮南绿豆饮品加工、华仪通榆风机制造、大安天能风电塔筒等重大项目落户集中区，有力地促进了县域经济发展。深入开展工业提速增效活动，全市规模以上工业企业增加到218户，占全年任务目标的105.8%；工业总产值、增加值增速分别达到24%、21%。万元GDP能耗下降4.5%，规模以上工业万元增加值能耗下降3.5%，完成了省下达的任务目标。强力推进工业企业招商重组，市直通业集团、白城纺织、麻纺织、红钻、金鹏齿轮等企业招商重组工作取得了一定成效。积极运作企业上市，白城中一精锻股份有限公司上市前期工作进展顺利，争取突破白城没有上市公司历史。启动实施“百户中小企业成长工程”，全市有60户企业被评为省级成长型中小企业，有7户企业进入了全省百强民营企业行列。预计全市民营经济主营业务收入完成257.3亿元，同比增长35.4%，民营经济实现了快速发展。

　　（五）注重城市开发管理，基础设施建设改造取得阶段性成果。加强中心城市建设，共筹措资金9.48亿元，实施市区基础设施建设改造八项工程：一是城市绿化工程，对市区25条街路、8处绿地、10条街路分车岛，以及公园、广场等进行了全面绿化，绿化面积增加到817万平方米，绿化覆盖率达到29.9%。二是道路改造工程，对市区内环和外环路面、分车岛边石进行了铺装和更换。这是白城市区环路建设八年来，第一次进行全面改造铺装。三是辽北路立交桥维修工程，对建设20年的辽北路立交桥路面、侧栏、底柱等进行了全面维修加固。四是热电厂供热管网改造配套工程，改造新建4座供热站，新增供热能力125万平方米。五是污水处理厂续建工程，完成了土建工程，以及二期嫩江路泵站设备安装。六是水源地保护工程，已纳入了松花江流域水源治理“十一五”规划，完成了前期工作。七是生活垃圾无害化处理工程，完成了氧化沟、二沉池等设施建设。八是商业网点规范化建设工程，对市区8条专业街、6个重点商品交易市场、1个商业中心进行了规划建设。加大了旅游、商贸等服务业扶持力度，预计全市服务业增加值完成103亿元，同比增长24%；社会消费品零售总额完成114亿元，同比增长19.4%。成功引进了全国前十位商业百强企业——长春欧亚集团，在白城建设欧亚购物中心项目。项目建成后将成为吉林西部规模最大、功能最全的现代购物中心和商业龙头企业。各县(市)也加强了城市基础设施建设，城市面貌发生了显著变化。

　　（六）坚持社会事业协调发展，和谐白城建设得到加强。加快推进科技进步，全市实施科技发展计划项目121个，其中有2个项目被列入国家创新基金计划，实现了我市在争取国家科技项目上的历史性突破。坚持优先发展教育，启动实施“新农村百校标准化建设工程”，4个县(市)职业教育中心均进入了省“百强校”行列。白城师范学院通过了国家教育部本科教学工作水平评估。白城医学高等专科学校通过了国家教育部高职高专人才培养水平评估。推进城乡医疗服务体系建设，全市农村三级卫生服务网络和城市社区医疗卫生服务体系建设全面展开，以手足口病为重点的疫病防控工作取得了明显成效。启动了白城中心医院整体迁建、白城中医院改扩建项目。构建公共文化服务体系，加快推进“农家书屋”和乡镇综合文化站建设，开发建设白城电影城。开展了纪念改革开放30周年各项活动。深入开展“全民健身与奥运同行”主题活动，成功举办了全市第四届田径运动会暨全民健身运动会。加强双拥工作，实现了全国双拥模范城“两连冠”目标。全面完成了人口控制计划，低生育水平持续稳定。广播电视、信息产业、民族宗教、人防、外事侨务、妇女儿童、老龄、残疾人、气象、地方志等事业都有了新的发展。全市国有粮食购销企业产权制度等改革基本完成。强化食品药品监管，“三鹿奶粉”事件得到有效处置。开展安全生产“隐患治理年”活动，全市各类安全事故同比减少23.9%。推进公安“三基”工程建设，强化治安巡逻防控工作，社会治安形势总体平稳。“五五”普法工作深入开展。经过艰苦努力，新建白城监狱犯人监舍6200平方米工程，已正式动工，明年将彻底解除监狱建筑隐患。落实信访责任制，深入开展大接访活动，北京奥运会、残奥会期间全市实现了非正常进京零上访目标。“5·12”四川汶川地震发生后，我市在全省率先组织规模空前的捐款捐物活动，全市累计接收捐赠款物1791万元。积极组建卫生防疫和公安消防队伍，赴四川汶川地震灾区开展工作，受到了省委、省政府表彰。认真做好对口支援四川省黑水县卡龙镇恢复重建工作。开展“支援灾区节约公用经费”活动，党政机关及事业单位公用经费一律压减年度预算的5%。

　　（七）努力保障和改善民生，承诺民生民利实事较好地完成。市政府年初承诺的十件民生民利实事全部完成。新增城镇就业4.3万人，开发公益性岗位750个，“零就业”家庭全部实现动态就业。农村劳务输出50.8万人次。城乡社会救助体系建设全面推进。城乡居民最低生活保障基本实现应保尽保。新型农村合作医疗参合人数达到101.7万人。启动城镇居民基本医疗保险工作，参保人数达到了33.4万人，完成省下达任务的111.4%。城镇职工基本医疗保险达到29.2万人。工伤保险人数达到11万人，生育保险人数达到12.1万人，均超额完成了省下达任务。改造了121万平方米的棚户区及危旧房，使8965户棚户区及危旧房居民乔迁新居。开工建设城市廉租房7万平方米。农村泥草房改造完成2.2万户。投资4.3亿元修建了952公里“村村通”等级公路，全市等级公路通村率达到了84.8%。加强113个贫困村脱贫工作，实现了3.67万农村贫困人口脱贫。实施农村饮水安全工程，累计使51万农村人口告别饮用苦咸水的历史。实施法律援助，为群众解决法律问题836件。

　　（八）推进政府管理制度创新，政府制度建设取得一定成效。我们把今年确定为政府“制度建设年”，市政府相继出台了22项各类规章制度，为政府实施科学行政、民主行政、依法行政奠定了基础。认真办理人大代表建议和政协委员提案，办复率均达到了100%。加强人事编制管理工作，事业编制实现了负增长。强化政府服务职责，行政审批权相对集中改革积极推进。加大行政效能建设力度，行政监督、审计监督不断加强。认真贯彻政府信息公开条例，政府信息公开全面推行。开展民主评议政行风活动，整治经济发展软环境取得了阶段性成果。全面落实党风廉政建设责任制，政府系统廉政建设取得了新成效。

　　总结今年工作，我们在特殊的经济形势、特别的艰难环境下，能够取得这样的发展成果，实属不易。这是在市委的领导下，市级各领导班子合力攻坚，全市上下共同努力的结果，也是我们坚持发展连续性、求真性、务实性的结果。在此，我代表白城市人民政府，向全市工人、农民、知识分子、各级干部，驻白部队、武警官兵和公安司法干警，中省直驻白单位，致以崇高的敬意！对人大、政协、各民主党派、工商联、人民团体和社会各界，离退休老领导、老同志给予政府工作的大力支持和热情帮助，表示衷心的感谢！

　　回顾今年以来工作，我们也清醒地认识到，当前全市经济社会发展和政府工作还存在许多的困难和问题，主要表现是：农民增收受到严重制约，统筹城乡发展任重而道远；受国际金融危机的影响，部分企业生产经营困难加重；国家财税政策新调整及民生刚性支出增多，我市财政收支矛盾进一步尖锐；信访形势依然严峻，维护社会稳定压力加大；以改善民生为重点的社会建设有待加强，政府社会管理和公共服务能力还需要强化；政府工作人员素质和依法行政能力需要进一步提高，一些部门不同程度地存在官僚主义、形式主义和铺张浪费等问题。对这些困难和问题，我们要高度重视，认真加以解决，使政府工作更加适应形势发展的新要求，更加顺应人民群众的新期待。

　　2009年是新中国成立60周年。针对国际金融危机冲击，做好2009年政府工作，对于维护全市改革发展稳定大局，加快推进白城崛起，具有十分重要的意义。

　　明年政府工作总体要求是：全面贯彻党的十七届三中全会、省委九届六次全会和全省经济工作会议精神，以邓小平理论和“三个代表”重要思想为指导，深入贯彻落实科学发展观，按照市委四届四次全会的部署，推进工业化、农业产业化、生态建设产业化进程，坚持保增长、扩内需、调结构，加大投资拉动力度，积极扩大消费需求，加快培育壮大优势特色产业，坚定不移地推进改革开放，努力保障和改善民生，促进白城经济社会又好又快发展。

　　综合考虑各种因素，2009年全市经济社会发展主要预期目标是：地区生产总值完成370亿元，按可比价格计算，同比增长21%；固定资产投资完成370亿元，同比增长41.2%；规模以上工业增加值完成42亿元，同比增长12%以上；全口径财政收入完成25亿元，力争同比增长18%；社会消费品零售总额完成135.7亿元，同比增长19%；城镇登记失业率控制在4.6%以内；城镇居民人均可支配收入14795元，同比增长10%；农民人均纯收入3900元，同比增长10%；人口自然增长率控制在4‰以内。

　　实现明年全市经济社会发展目标，政府工作要全面贯彻落实扩大内需、促进经济增长政策措施，把保持经济平稳较快发展作为首要任务，在保投资、保增长、保民生、保稳定上下功夫，做到“四抓四促”：坚持抓投资促消费，强化投资主体作用，着力保持经济平稳较快发展；坚持抓增量促存量，发展资源型产业，着力做大做强优势特色产业；坚持抓改革促开放，深化重点领域和关键环节改革，着力提高招商引资质量和水平；坚持抓民生促稳定，切实解决涉及群众利益的难点热点问题，着力加强和谐白城建设。

　　明年，我们要重点抓好以下八个方面工作：

　　（一）抢抓国家重大政策机遇，推进我市基础设施建设跨上新台阶。牢牢抓住国家扩大内需、促进经济增长的政策机遇，重点组织实施五大工程：一是水利工程。加快引嫩入白、大安灌区、洮儿河灌区续建配套与节水改造、月亮泡蓄滞洪区、分洪入向除险加固“五大水利工程”在建项目建设进度，抓好节水灌溉工程建设，实施向海、莫莫格自然保护区生态移民工程。二是公路工程。全面开工建设投资46亿元的国道珲乌线大安至石头井子高速公路。白城绕越线实现竣工通车。尽快开工建设齐齐哈尔嫩江至通辽嫩通高速公路白城段工程。抓紧推进黑龙江铁力至内蒙古科左前旗高速公路白城段，以及泰大、大通、长扎、舍突等四条县级公路升级为省级二级公路的前期工作，争取尽早立项、开工建设。抓好“村村通”等级公路1430公里工程建设。三是铁路工程。启动实施白阿线白城至乌兰浩特、长白线长春至白城增建二线铁路建设工程，抓好白城火车站扩建，加快推进市区金辉街等县(市)公铁立交桥建设。四是机场工程。白城民航机场已纳入了我省“八大”重点基础设施建设工程之一。加大前期工作推进力度，争取明年开工建设。五是城市公共设施工程。加强城市交通、供水、供热、供气等设施配套建设。搞好白城货运仓储站、欧亚购物中心、海明路商业步行街路面改造、全民健身活动中心等项目建设，使城市基础设施承载功能实现新发展。

　　（二）加大投资拉动力度，滚动实施好重大项目建设。充分发挥投资带消费、促增长的主导作用，在今年基础上，明年我们要滚动实施千万元以上“三个一百”重大项目建设，总投资2451.2亿元。其中在建项目建设100个，总投资493.3亿元；新开工项目100个，总投资417.9亿元；前期工作项目100个，总投资1540亿元。投资超亿元项目198个，其中10亿元以上项目52个。抢抓国家加大基础设施、民生工程、生态环境建设等方面投入的难得历史机遇，千方百计争取资金、争取项目。要成立重大项目推进组??重点规划好投资10亿元以上重大项目，争取纳入国家新增投资建设计划。坚持一手抓向上争取、一手抓招商引资，创新招商引资方式方法，大力发扬千辛万苦、千山万水、千言万语、千难万险的“四千四万”精神，在引进战略投资者、推动我市优势特色产业发展上取得新的突破。

　　（三）着力打造吉林西部能源城市，做大做强火电、风电产业。积极推进中电投白城发电公司一期工程2×66万千瓦项目建设速度，加快做好二期工程2×100万千瓦项目前期工作，为实施三期工程2×100万千瓦项目创造条件，使中电投白城发电公司总装机容量达到532万千瓦。加快白城500千伏输变电工程建设进度，确保明年竣工运营。全力做好白城热电厂2×20万千瓦扩建项目的核准工作，加快开工建设步伐。搞好总投资10亿元的通榆500千伏输变电工程项目建设。协调推进分别投资10亿元的大安、镇赉500千伏输变电工程，以及分别投资160亿元的大安、镇赉火电项目前期工作。抓好农村电网改造。明年，全市风电开发建设装机要新增100万千瓦、总量突破200万千瓦，并争取纳入国家千万千瓦级大型风电场规划。着力发展壮大风电设备制造业，加强洮北风电塔筒、吉林名门洮南塔筒扩建改造、白城天奇新能源风电设备、华仪通榆50万千瓦风机制造等重点项目建设。抓好大安、镇赉、洮北油气增产开发，协调好通榆石油勘探，促进洮南煤炭、铜矿开采，使全市形成绿色能源产业突出、其他能源产业深入发展的局面。

　　（四）建设国家级生态农业示范区，强力推进粮食增产工程建设。加快实施粮食增产工程，把我市建成全省水田大市、杂粮杂豆和油料大市。加速推进土地开发整理、标准粮田建设、空中云水资源开发、生态建设等11个工程、22个项目建设进程，使我市成为吉林省增产百亿斤商品粮主体区。力争用三年时间，全市水田面积达到350万亩，成为全省水田第一大市。发展特色农业，加强杂粮杂豆和油料基地建设，制定全市杂粮杂豆和油料生产规划，形成板块式区域化生产格局，使我市成为全省乃至全国重要的杂粮杂豆和油料生产基地。抓好镇赉、大安生态农业示范区项目，建设国家一流生态农业示范区。规划启动向海现代生态经济示范项目区建设。加快发展生态产业，围绕种植100万亩桑树、150万亩山杏、200万亩白刺的规划目标，积极扩大种植面积，推进“三大生态产业工程”建设。加强100万亩芦苇基地建设。搞好200万亩碱地脱硫废渣改造项目。加快现代烟草农业发展。做好土地利用总体规划修编前期工作。着力促进全市生态农业、特色农业发展，形成粮食增产、生态改善良性发展的格局。

　　（五）不断发展壮大各级工业园区，加快推进新型工业化进程。发挥白城工业园区引领带动作用，抓好标准街路、污水泵站及管网工程、综合服务中心、商业住宅配套区等基础设施配套建设。坚持一手抓建成项目达产达效，一手抓重大项目强力引进，推进白城工业园区快发展、大发展。明年，白城工业园区工业产值要达到25亿元以上。突出办好县(市)工业集中区，加快工业集中区基础设施配套建设，努力争取省里关于县(市)工业集中区建设以奖代补的政策。坚持集中摆放重大工业项目，做到要素集聚、产业集中、企业集群发展。加强白城经济开发区、查干浩特旅游经济开发区、白城民营经济开发区和大安经济开发区建设，坚持以项目建设为重、以培育特色产业为主，在做大做强上下功夫。落实省委、省政府实施工业产业跃升计划，着力培育我市能源和农产品加工两大支柱产业。利用国家促进企业兼并重组政策，积极推进工业企业兼并重组，抓好白纺、金鹏齿轮、国宏公司等市直17户企业招商重组工作。深入开展工业提速增效和“工业项目年”活动，抓住国家实施增值税转型改革的有利时机，实施一批重大技改项目。明年，全市工业投资和技术改造投资分别完成150亿元、60亿元，同比分别增长36.4%、9.1%，重点行业、重点项目开工率达到80%以上。必保完成省政府下达的节能减排任务指标。大力扶持中小企业，加强创业孵化基地建设，推进民营经济加快发展。

　　（六）坚持统筹城乡发展，加快农村改革发展步伐。坚持农村基本经营制度，稳定现有土地承包关系长久不变。按照依法自愿有偿的原则，促进土地经营权有序流转。围绕建设牧业大市、特色农产品加工大市，启动10万头奶牛标准化规模养殖小区、10万亩草基地、50万张皮革深加工产业开发、10万吨秸秆生物饲料加工项目建设。实施农产品加工业提升战略，重点抓好具有白城特色的玉米、水稻、油葵、辣椒、绿豆、蓖麻、汉麻、燕麦和花生等农产品加工项目建设。全力推进新加坡益海嘉里粮油食品加工、洮北吉鸭牧业1000万只樱桃谷肉鸭加工、镇赉飞鹤乳制品加工等重点项目建设，形成特色农畜产品加工企业集群。促进农业科技园区建设和庭院经济发展，大力发展棚膜蔬菜产业，抓好国家农业标准化示范区项目。加快小城镇建设，进一步完善小城镇发展规划，培育和壮大一批工业强镇、商贸大镇、旅游名镇、民族集镇等。着力发展劳务经济，加快农村劳动力转移就业。强化现代服务业发展，打造特色旅游品牌。扎实推进新农村建设，大力发展农村社会事业，加强农村基础设施和公共设施建设，切实改变城乡面貌。

　　（七）努力保障和改善民生，切实把发展成果惠及群众。明年，围绕改善民生，我们要集中力量为群众办好八件实事：一是新增城镇就业3.7万人，开发公益性岗位保持在1万人的就业规模。二是新型农村合作医疗参合率力争在现有基础上再提高5%，城镇居民基本医疗保险参保人数达到30万人，城镇职工基本医疗保险参保人数达到30万人。改善医疗卫生条件，加快白城中心医院整体迁建、白城中医院改扩建项目建设，抓好乡镇、农林牧渔场卫生院改造。三是教育投入1.05亿元，全部免除城乡义务教育阶段21.4万名学生学杂费，对城市低保家庭学生免费提供教科书。搞好城乡中小学校舍改造。四是实施安居工程，改造棚户区及危旧房150万平方米以上，建设城市廉租房6万平方米，改造农村泥草房2.35万户。五是实施扶贫开发推进计划，实现2.75万农村贫困人口脱贫。六是解决农村1.1万人饮水安全问题，全面完成农村饮水安全工程改造任务。七是新建“农家书屋”100个，建设农村文化大院100个。八是对城市6万户有线电视实施数字化整体转换，全面完成农村广播电视“村村通”工程。同时，加快建设市区污水和垃圾处理工程，加强市区道路改造。以上这些民生实事，市政府要全力践行承诺，必保如期兑现。坚持统筹发展社会事业，抓住国家加快科技、医疗卫生、文化教育等事业发展的机遇，全力实施一批重大科技发展项目；积极稳妥推进医疗卫生体制改革，完善农村三级卫生服务网络和城市社区卫生服务体系，加强疾病防控和医疗救助基础设施建设；构建公共文化服务体系，加快白城博物馆、图书馆、群众艺术馆等项目建设，加大城乡图书馆、文化馆以及乡镇文化站等文化设施建设力度；推进“新农村百校标准化工程”建设进程，加强中等职业教育基础建设，做好白城职业技术学院教学水平评估前期准备工作。深入开展全民健身活动。坚持计划生育基本国策，稳定低生育水平，统筹解决人口问题。加强国防建设，搞好双拥工作。加强人事人才工作。发展妇女、儿童、老龄事业。积极主动做好新形势下的宗教和侨务工作。抓好信息产业、气象、地震、人防、地方志等工作。

　　（八）加快政府职能转变进程，努力建设人民满意政府。坚持人民政府为人民的施政理念，强化服务建设，充分发挥政务大厅服务功能，全面推进行政审批权相对集中改革，切实把政府信息公开落到实处，大力整治和建设经济发展软环境，深入开展市长公开电话进社区、进乡村、进企业活动。强化制度建设，坚持依法行政，全面贯彻落实国务院关于加强市(县)政府依法行政的决定，严格执行行政执法责任制和执法过错追究制。强化社会建设，加强社会治安综合治理，深入开展“平安白城”创建活动；认真贯彻突发事件应对法，有效预防和处置突发公共事件；高度重视安全生产和食品安全工作；全面落实信访工作领导责任制和责任追究制，完善解决信访问题保障机制，健全矛盾纠纷排查化解长效机制；加快推进依法治市进程，深入开展“法律六进”活动。主动接受人大的法律监督、工作监督和政协的民主监督，加强舆论和群众监督。认真办理人大代表建议和政协委员提案。强化效能建设，突出政府执行力建设，建立和完善重点工作目标责任制，全面开展政府绩效评估工作，推进绩效审计，实行行政问责制。推进重点领域和关键环节改革，抓好厂办大集体、集体林权、成品油价格和税费等方面改革，全面完成政府机构改革任务。积极做好吉林银行白城分行筹建工作，力争明年初挂牌运营。深入开展厉行节约、压缩开支活动，抓好增收节支工作，实现行政公用经费“零增长”目标。加强反腐倡廉建设，严格执行党风廉政建设责任制。开展好学习实践科学发展观活动，从市政府领导做起，带头学习实践科学发展观，做到说实话、办实事、讲实效，实实在在谋发展，勤勤恳恳干事业，做出经得起实践、人民、历史检验的实绩。

　　各位代表！展望未来的白城，充满希望，催人奋进。让我们在市委的领导下，全面贯彻落实科学发展观，凝聚全市人民的智慧和力量，抢抓机遇，锐意进取，为实现白城经济崛起、社会和谐、人民富裕而努力奋斗！
